# Ambassadors of hope, research pioneers and agents of change—individuals’ expectations and experiences of taking part in a randomised trial of an innovative health technology: longitudinal qualitative study

**DOI:** 10.1186/s13063-019-3373-9

**Published:** 2019-05-27

**Authors:** Julia Lawton, Maxine Blackburn, Jenna P. Breckenridge, Nina Hallowell, Conor Farrington, David Rankin

**Affiliations:** 1 Usher Institute of Population Health Sciences and Informatics, The University of Edinburgh, Edinburgh, UK; 2 School of Nursing and Health Sciences, University of Dundee, Dundee, UK; 3 Wellcome Centre for Ethics and Humanities and the Ethox Centre, Nuffield Department of Population Health, Big Data Institute, University of Oxford, Oxford, UK; 4 Cambridge Centre for Health Services Research, University of Cambridge, Cambridge, UK

**Keywords:** Trial participation, Trial effects, Diabetes, Qualitative research, Clinical trial, Technology, Medical device

## Abstract

**Background:**

While a growing body of research has explored why people take part in clinical trials, this research has not considered how people’s understandings, motivations and agendas might influence their conduct during a trial. This is an important area of enquiry because it is now widely recognised that an intervention might lead to different clinical outcomes when delivered as part of a trial than when implemented in routine clinical practice; however, the reasons for this are not fully understood.

**Methods/design:**

We interviewed 24 individuals who took part in a trial of an innovative health technology under development for people with type 1 diabetes which automatically regulates blood glucose: the closed-loop system. Participants were interviewed following randomisation to a closed-loop and at trial closeout.

**Results:**

Participants provided complex agendas for taking part in which altruistic and self-interested considerations were often inseparable. Many described belonging to a wider diabetes community and being beneficiaries of others’ participation in research and how this had given rise to attendant citizenship obligations. Participants also shared the excitement and pride they experienced from contributing to research which situated them at the forefront of technological innovation and enabled them to present themselves to others, by virtue of their trial participation, as ambassadors of hope and research pioneers. Given their desire to support the progression of a potentially life-changing technology, and be part of that innovation, participants, at follow-up, described having made extra effort during the trial. Specifically, participants described having been more focused on their diabetes management to help create conditions in which the closed-loop could work most effectively to optimize their blood glucose control.

**Conclusions:**

Our findings contribute a new dimension to understandings of trial effects; specifically, we argue that, to aid interpretation of trial outcomes, participants’ understandings and motivations for participation need to be considered. We highlight the potential pertinence of our findings in the contemporary era of bio-citizenship where, increasingly, people are driving research agendas and see themselves as co-producers of knowledge. We also recommend a new concept be introduced into the literature—‘the altruselfish agenda’—to recognise potential inseparability of self-interested and altruistic motivations.

**Trial registration:**

ClinicalTrials.gov, NCT02523131. Registered on 14 August 2015.

## Background

Clinical trials depend on volunteers who might not derive personal health benefit from taking part. Hence, it has traditionally been assumed that altruism—that is, a willingness to help others and contribute to the public good—is a key driver of participation [1]. However, as a growing body of qualitative research has made apparent, individuals’ reasons for participating in trials may be multifaceted. Specifically, individuals tend to present self-interested agendas alongside altruistic considerations [2–5]. The former might be a hope or expectation of receiving individualized clinical treatment, despite trial research being hypothesis rather than needs driven [1, 5]. People may also choose to take part in trials because of perceived opportunities for personal therapeutic benefit arising from ‘incidental’ features of the research, such as increased monitoring and access to health professionals—what has been termed ‘therapeutic appropriation’ [6]. Given that self-interested and altruistic considerations appear to feature in most individuals’ decision-making, some authors have used caveated terminology to better capture their motivations and agendas. This includes the concept of ‘weak altruism’ used by Canvin and Jacoby [5] to describe a situation wherein epilepsy trial participants were “happy to help others but only where they could also help themselves” (p10). McCann et al. [1], similarly, used the term ‘conditional altruism’ to describe how the people in their study were willing to help others with the proviso that trial participation would also benefit themselves.

The above studies have made an important contribution to the literature, primarily through offering insights to help future trial recruitment [1, 2, 4, 5] or by raising and/or addressing concerns about whether people are making fully informed decisions about taking part [7–9]. Currently missing from this literature is consideration of how people’s understandings and motivations for taking part might influence their conduct during a trial. This is an important area of enquiry because it is now recognised that an intervention may be delivered and received differently within the context of a trial and, hence, might lead to different outcomes than when implemented in routine clinical practice [10, 11]. Indeed, the value of undertaking qualitative (and mixed-methods) research within trials has been emphasised for this reason [10, 12]. To date, however, most qualitative studies have focused on staff perspectives and experiences, as a recent overview of the literature makes apparent [12]. When trial participants’ perspectives have been considered, studies have tended to focus on their likes and dislikes of the intervention [13], mechanisms and mediators of change [14] and reasons for (non) adherence to the treatments/interventions under investigation [15, 16], without the potential impact of being in a trial being taken into account.

In this paper, we report findings from a longitudinal qualitative study undertaken with people who were involved in a randomized controlled trial of a diabetes technology, currently under development, called a closed-loop system—which is sometimes also called an artificial pancreas. This three month trial [17], which involved both adults and young people, built on previous investigations undertaken by the same team and sought to test the acceptability and efficacy of the closed-loop system (as compared to an open-loop system) in the management of type 1 diabetes, which is a chronic disease which occurs when the pancreas is unable to produce insulin. The closed-loop system comprised an insulin pump, a continuous glucose monitor and a computer-based algorithm which resided in a separate handheld device and which translated, in real-time, information received from the continuous glucose monitor (which measured interstitial blood glucose every 5–7 min) in order to compute the amount of insulin delivered by the pump. Hence, the intended purpose of the closed-loop was to improve blood glucose control as well as reducing the burden of diabetes management [17]. Indeed, in the trial information sheet, potential participants were advised that taking part “could help further development and refinement of closed-loop systems that can control blood sugar in people with diabetes.”

A key aim of our interview study was to understand and explore how people used the closed-loop to support diabetes management in order to aid interpretation of trial outcomes and provide recommendations for training and support which could be given to future users to help promote optimal use of the technology [18, 19]. As part of this investigation, we explored people’s reasons for taking part in the trial as well as their actual experiences of using the closed-loop system during the trial. As we describe in this paper, the findings from this aspect of our study not only prompted us to problematise use of dichotomous categories such as ‘self-interest’ and ‘altruism’, but also to contribute literature on clinical trials by considering how participants’ understandings of the trial and their complex and interweaving agendas for taking part could have profound implications for their conduct during the trial.

## Methods/design

Semi-structured interviews were used as these afforded flexibility for participants to raise and discuss issues they perceived as salient, including those unforeseen at the study’s outset [20]. These interviews were undertaken at two time-points: just after participants had been randomized to a closed-loop and following their closeout from the trial 3 months later. Data collection and analysis took place concurrently, enabling findings from early interviews to iteratively inform areas explored in later ones in line with an inductive approach. As detailed elsewhere [18], the study was informed by Normalization Process Theory [21], an epistemological position which recognises that there may be unintended consequences arising from using a new technology, and that its use may be influenced by personal and contextual factors which need to be captured and explored as part of the data collection and analysis process.

### Sample and recruitment

Three groups of individuals were recruited into the interview study by trial staff in the four participating sites in the United Kingdom (Cambridge, Edinburgh, Manchester and Leeds) using an opt-in procedure: adult trial participants (18+ years), adolescent trial participants (13–17 years); and, parents/caregivers of trial participants aged 13–15 years and 12 years and under. The decision to interview parents/caregivers of trial participants aged ≤ 12 years and those aged 13–15 years was made because, in pre-teenage groups, parents take overarching responsibility for decision-making and diabetes management tasks, while sharing these responsibilities with adolescents. At the time of recruitment and before commencement of their interviews, it was made clear to all participants that the interview study was being conducted by an independent team of experienced qualitative (non-clinical) researchers and that all information shared would be kept strictly confidential. Recruitment continued until there was adequate representation of the different participant groups in the final sample and data saturation occurred; that is, until no new findings were identified in new data collected.

MB, an experienced interviewer, conducted the interviews at a time and location of participants’ choosing (mostly in their own homes) using topic guides which were developed in light of literature reviews, inputs from parent and patient representatives and members of the trial’s clinical team, and revised in light of emerging findings (see above). Key areas explored in the interviews which are relevant to the reporting in this paper are provided in Table 1.

**Table 1:** key areas explored in the interviews

Baseline interview • Background information about study participant, including: who they live with and what they do (everyday work/school and family life); details about the child trial participant with type 1 diabetes if the interviewee is a parent • Experience of diabetes management prior to the trial, including: previous regimen used; any challenges and difficulties encountered undertaking diabetes management tasks and maintaining/achieving optimal blood glucose control; role and involvement of parents of child trial participants in undertaking diabetes management tasks ○ Probe to explore: food choices, experiences of counting carbohydrates, frequency of blood glucose testing, experiences of determining and administering insulin doses, use of blood glucose readings, etc. • Knowledge and understanding of closed-loop technology (e.g. how the closed-loop works and what it is meant to do, likes and dislikes); sources of this knowledge • Previous experiences of taking part in trials/closed-loop research • Experiences of being recruited into the trial; understandings of the trial and its purpose; reasons for agreeing to take part (or supporting child’s decision to take part) • Reactions to discovering oneself/one’s child had been randomised to the closed-loop system • Hopes, expectations and concerns regarding using the closed-loop during the trial	
Follow-up interviews • Any changes in personal circumstances (e.g. employment, home set up, lifestyle), since previous interview • Experiences of using the closed-loop system during the trial, including likes and dislikes of using the technology; key challenges encountered using the closed-loop system; comparisons with previous regimen; reasons for using/not using the technology in the ways recommended by trial staff; parents’ views about children operating the closed-loop system • Experiences undertaking diabetes management tasks during the trial ○ Probe to explore reasons for any changes in key diabetes self-management tasks and behaviours since previous interview (e.g. changes in dietary choices, frequency of and views about blood glucose testing, reviewing blood glucose data, etc.) • Participants’ or parents’ level of involvement in managing diabetes while using the closed-loop system and how this compares with using previous regimen ○ Probe to explore whether participants’/parents’ focus on diabetes has changed in any way while taking part in the trial, and why • Perceptions and understandings of how the closed-loop system worked and how it affected one’s (or one’s child’s) blood glucose control • Views about impact of using the closed-loop system on quality of life • Input and support sought and received from staff during the trial; reasons for contacting staff for support • Views about how the technology could be improved, and why • Hopes and expectations regarding the future developments in closed-loop systems and other diabetes-related technology	

Interviews took place between July 2016 and August 2017, typically lasted 1–2 h and were digitally recorded and transcribed in full. Data were analysed by four experienced qualitative researchers (JL, MB, DR and JPB) using a thematic approach involving cross-comparison of all interviews to identify recurrent themes. Each participant’s baseline interview was also read in conjunction with their follow-up interview to identify continuities and changes in their accounts over time, and compare expectations with actual experiences of trial participation. The researchers undertook initial data analyses independently and wrote separate reports before meeting to discuss their interpretations and reach agreement on key themes. A coding frame was then developed which captured these themes and contextual information needed to aid data interpretation. Nvivo Version 10 (QSR International Pty Ltd, Doncaster, Victoria, Australia), a qualitative software package, was used to facilitate data coding and retrieval and coded datasets were subjected to further analyses to allow more nuanced interpretations of the data to be developed. To safeguard anonymity, unique (gender neutral) identifiers are used below.

## Results

All participants approached opted-in to the study. See Table 2 for details of the sample which comprised ten adult trial participants (aged 18+ years), five adolescent trial participants (aged 13–17 years) and nine parents. Below, we begin by reporting participants’ complex agendas for taking part before considering how these appeared to influence their conduct during the trial. As all of the main findings cut across the sample, our reporting has not been separated out according to participant groups (e.g. adults, adolescents and parents) and whether individuals had prior experience of taking part in closed-loop research (Table 2).

**Table 2:** Demographic characteristics of study participants

Characteristic	N	%	Mean ± SD and range
Adults (*n* = 10)			
Female	5	50.0	
Age (years)			39.6 ± 10.6, range 28–65
Occupation			
Professional	5	50.0	
Semi-skilled	4	40.0	
Manual	–	–	
Carer/not working	1	10.0	
Previous experience of using a closed loop	3	30.0	
Adolescents (*n* = 5)			
Female	2	40.0	
Age—all children			15.4 ± 1.5, range 13–17
Education			
Secondary school	2	40.0	
Higher education	3	60.0	
Previous experience of using a closed loop	3	60.0	
Parents of child participants (*n* = 9)*			
Female	5	55.6	
Age			44.6 ± 5.6, range 35–53
Occupation			
Professional	5	55.6	
Semi-skilled	2	22.2	
Manual	1	11.1	
Carer/not working	1	11.1	
Previous experience of using a closed loop	3	33.3	

*This includes parents who represented children aged ≤ 12 years (*n* = 5) and parents of adolescents aged 13–15 (*n* = 4). In one instance, both parents of a child aged 13–15 years participated in an interview

### Self-interested agendas: therapeutic appropriation and incidental benefits

For a minority, reasons for taking part in the trial were driven by self-interested considerations. Such individuals discussed the personal health benefits they hoped to gain from having access to continuous glucose monitoring data (available to individuals in both trial arms) as they hoped this could be used to help improve their blood glucose control both during and after the trial, with some noting that this potential benefit had been pointed out at the time of recruitment. This included Adult 6 who described how they had:

“hope [d] to improve my diabetic control, for my own health and benefit … and I’m quite keen on having a second child, and I was thinking, if I have four months of [continuous glucose monitoring] data, that’s properly going to help my control as well.”

Participants’ enthusiasm for taking part in the trial was also fuelled by the potential opportunity it presented to use the latest version of the closed-loop. This included Adult 3 who highlighted how their own curiosity had been the main driver: “I’m interested in how it [closed-loop] works, how quickly the algorithms work and just the science bit of it.” Adolescent 2, similarly, noted how the possibility of experiencing closed-loop technology first-hand had influenced their decision-making because:

“closed-loop is like the most exciting thing in the diabetes world at the moment ... obviously some people don’t even get the slightest opportunity to try it, especially not for such a long period of time. So it’s just sort of like, well yeah, I’m just gonna do it, because I’ve got this opportunity and it’s really sort of special.”

Others highlighted additional benefits they hoped to gain, albeit these were not normally central drivers or considerations. Adult 8, for instance, described how they had become a “little bit slapdash” with their diabetes self-management and had hoped that trial participation, and regular contact with trial staff, would force them to “refocus on my diabetes again … I needed this kick up the backside to do it really.” According to Parent 7, increased contact with clinical staff was also an incentive for their child’s participation; in this particular case because of heightened feelings of self-worth arising from being the centre of trial staff’s attention:

“He … doesn’t ever feel really special. He obviously feels very different. So for [child’s name], they made him feel like an absolute king when he first visited [names city]. And I think that’s nice for him. And I think he likes that feeling.”

### Altruistic agendas: helping others

A minority, at the other extreme, presented ostensibly altruistic motivations by stressing that they expected to gain little or no personal benefit from taking part. This included Adult 1 who described how:

“I’ve had diabetes for 36 years, I’m more than happy to help out with these trials for the simple reason that if people don’t, it’s not gonna progress … and if it all works, the technology will progress, it will become cheaper, so it can be put out on the open market, and then hopefully youngsters, that kind of technology would be there for them.”

Indeed, reinforcing their suggestion that altruism had been their central motivation, Adult 1 also noted how if, by virtue of randomization, they had not been in the closed-loop arm of the trial:

“I wouldn’t have had a problem with it, ‘cause they need the information in order to marry up against the closed-loop, you know, you can’t just test one side, you’ve got to go test the other. So, you know, both are equally valuable.”

While Adult 1 was keen to present themselves as a “willing volunteer for these kinds of studies” this individual, like some others, also alluded to a sense of obligation; albeit, none reported any pressure from staff to take part. On the contrary, many described how their involvement in diabetes groups and online forums together with reading articles and diabetes magazines had generated or reinforced a sense of belonging to “a wider diabetes community” (Adult 4), one which gave rise to attendant obligations and responsibilities. As such participants noted, access to these groups and information in the media had not only enabled them to keep up to date with developments in closed-loop and other diabetes technologies, it had also fostered an awareness that they were beneficiaries of treatments made possible by others’ participation in earlier diabetes research. This included Adult 9, who noted how: “people had to test the first insulin, people had to test the first pumps, people had to do this to get to where we’re at now. Somebody’s always got to take that leap of faith.” A similar observation was made by Adult 1 who described how “if somebody didn’t stick up their hand and say ‘yes, I’m willing to try insulin’, that wouldn’t have progressed.” In making these kinds of observations, participants, including Parent 1, also noted the hypocrisy of benefiting from diabetes treatments without being willing to reciprocate and contribute to research by putting themselves or their child forward:

“I think the way that things are moving with the research and things, I think it’d be sort of hypocritical to say you know: let other people do this trial. And then when everything’s done, oh yeah, then we’ll have one of those please.”

A similar perspective was reported by Adult 4 who had learned about developments in closed-loop technology from a Facebook group, and who discussed how:

“I’m naturally kind of happy to take part in trials particularly around diabetes, because I get an awful lot out of the NHS and I think it’s only fair—and if I’m expecting a cure—I’m gonna want people to have done this trial.”

Indeed, when asked specifically why they had agreed to take part, Adult 4 responded:

“There’s my own personal short-term answer which is I want the [continuous glucose monitoring] data. But there’s the larger picture of an improved treatment for the diabetic community. Erm, to be honest it’s on the future diabetic community probably … there’s always at the back of my mind, I was four when I became diabetic, my daughter is now four.”

Hence, even when individuals emphasised ostensibly altruistic motivations, most also alluded to self-interested considerations, whether this be gaining access to data and support from staff which might help improve their own diabetes self-management or, in Adult 4’s case, supporting research which sought to progress a technology from which they or their own offspring might ultimately benefit.

### Interweaving and inseparable agendas: research pioneers and ambassadors of hope

As the above accounts suggest, while self-interested and altruistic elements were apparent to varying degrees in all accounts, in many cases these motivations appeared to be so inextricably interwoven that they were inseparable. This inseparability became particularly apparent when participants described the excitement they had experienced, and personal satisfaction they had gained, from being given the opportunity to be involved in what they saw as a pioneering study which could be “a proper game changer” (Adult 6). This included Adolescent 2 and Adult 8, who, like others, suggested that being part of closed-loop research was much more exciting and satisfying than learning about developments in the technology second-hand:

“I thought it was quite exciting. And I just thought: well this could change future technology for other people. So I just thought: yeah I’ll be part of that.” (Adolescent 2)

“you know, you’re always hearing about things which are in development, which are going to go forward, but to actually get to the point where, ‘ok, I’m taking part in a trial’ feels like you’re a bit closer to that and you can help with that progress, and you’re not just reading about it in the paper.” (Adult 8)

The excitement arising from taking part in a trial which could help advance closed-loop technology was also made only too apparent when Adult 9 shared their reaction to discovering they had been randomized to the closed-loop arm:

“I was pretty much useless at work, I was like a child at Christmas. It is that much of a big deal, it really is … cause if it works and it goes well, and it becomes a product which can be supplied to the masses, it’s gonna absolutely change lives, and I am going to be part of that.”

Indeed, as many individuals, including Adult 9, noted, trial participation not only presented an exciting and privileged opportunity to be directly situated at the forefront of technological developments, but also to be part of research which, ultimately, could change other people’s lives. Such an issue became apparent when Adolescent 1’s parents, in a joint interview, reflected upon why they had supported their child’s decision to take part in the current and previous closed-loop studies:

(Mum) “I think for him he sees the trials as a positive part of his diabetes ‘cause it’s kind of hope and technology and new things. So I think the trial is not only hope because, for him, it seems very, you know, like romantic, helping others. But it’s not only that. For himself he sees it as the exciting part of having diabetes … (Dad) he has come out saying: ‘how many people am I helping dad?’ ‘How many do you think [child’s name]? Millions? There are millions of people in the world with diabetes [child’s name].’ And that’s made him feel good.”

Connected to their desire to contribute to research which could help other people, participants described gaining satisfaction from being able to present themselves as research pioneers and ambassadors of hope within their diabetes networks. A particularly pertinent example was provided by Adolescent 5, who had already taken part in several closed-loop studies. While keen to emphasise a selfless agenda wherein, as they noted, “it’s unlikely I’ll have one when it first comes out”, Adolescent 5 also related an anecdote which made apparent that sharing their experiences of participating in closed-loop research gave rise to feelings of intense personal gratification and pride:

“I went to a diabetes kind of get-together thing... And this woman had a young son with diabetes and she was scared overnight because she thought that he would have a hypo and possibly die ‘cause he wouldn’t wake up. And just to think that this stuff [closed-loop technology] is actually so close that it’ll help so many people and that you’re a part of that... and you can kind of be able to tell people who are kind of a bit down, ‘this is—it’s soon. You’ll—you will have—this technology is working. You’ll be able to do—that you’ll be able to have no real worries soon.’ It’s a great experience.”

The satisfaction arising from sharing one’s own or one’s child’s experiences of contributing to closed-loop research was equally apparent when, in her interview, Parent 6 related a separate but similar anecdote:

“And I bumped into a lady when [name’s child] was on one of them [closed-loop], whose daughter had only just been diagnosed … And she was saying yeah ‘there isn’t light at the end of the tunnel’, and I said to her: ‘no no, no, there is light at the end of the tunnel, you know, you’ve not got to live with the injections forever and the constant worry when they’re that little of hypos’ … because they’re doing so much research. And they’ve got so much funding to do this … so, yeah, it’s nice to know that in some way we’re helping bring that day closer.”

### Agents of change

While participants thus valued opportunities to be involved in cutting-edge research, many also indicated that their excitement and motivation for taking part in the trial had been heightened by their perception that they could be active agents in the research process. This included Adult 7 who described how they had decided to take part because, “I wanted to be one of the first people to sort of assist in bringing it to reality in this country” and Adult 10 who suggested that “that’s why I’m taking part, hopefully this’ll help these things become a reality … I hope that in my small way I can—my data will push, advance the technology forwards.” Indeed, as Adult 10’s comments make apparent, many participants shared a perception and understanding that their own (or their child’s) participation could produce data which could have a tangible impact on the trial’s outcomes. In some cases, this perception appeared to have arisen from their experiences of taking part in, or reading about, previous, small-scale closed-loop studies and noting, as Adult 1 did, that the technology had evolved in very obvious ways as a result of this earlier research:

“So the first trial I did was like night-time only … and there was only 16 of us in that. And the technology was different and the computer you had was huge as well in comparison. And as a result of our study they did the one day one [another trial], a computer and a mobile phone, and then there is this one, basically a mobile phone.”

Others, including Adult 6, who described their understanding of the trial as being “ultimately to get it licensed”, also pointed to what they saw as the relatively small numbers involved in the current study. As this individual speculated, this potentially meant that their own (and others’) data could be very influential: “it’s quite small numbers of patients, isn’t it. Cause there’s 40 on this trial. Assuming it’s a 50/50 randomization, that’s only 20 patients to show it works and that it is safe.”

Indeed, with such an awareness and understanding in mind, another participant, Adult 7, who described themselves as having “a bit of knowledge around the area” due to working in a technology-related field, and saw the current trial as a proof of concept study, speculated that they might have been approached to take part because they were particularly well placed to help evidence the potential efficacy of the closed-loop system:

“that is why [local PI] pulled on me … with my knowledge and scientific background I could potentially help to bring it, to make it into a reality for more people who don’t have the knowledge or ability to do this sort of thing.”

### Impact of motivations and agendas on the participants’ conduct during the trial

As the above accounts suggest, most participants were heavily invested in the trial insofar as they really wanted it to help progress closed-loop technology and also because of their perception that their own involvement could help support this process. Indeed, such a goal was explicitly articulated by Parent 6 who saw their child’s participation as a way of: “help [ing] them with their data really, getting as much data as they can … so they can prove this really helps and then they’ll get more funding to do the next stage, so eventually it will become a licensed product.”

As described earlier, follow-up interviews afforded an opportunity to explore whether participants’ understandings of the trial and their agendas for taking part had any implications for their trial conduct. In keeping with their initial agendas, and their understandings that the technology would need further refinement before being ready for commercial use, participants described having put up with a level of inconvenience during the trial which, they suggested, would have been difficult to sustain in non-trial settings. As well as having had to attend frequent clinic appointments and upload data for the trial team on a weekly basis, participants described having had to use a prototype device, which was the size of a large mobile phone, and which they had found heavy and inconvenient to carry around. Participants also noted how they had persevered with using this technology and had made effort to keep their devices charged and in close proximity (so that the signal between the handheld device, the insulin pump and the continuous glucose monitor was not lost) because of their understanding that the trial first needed to “prove that a closed-loop can be done [developed] and can be done safely before moving on to the next stage” as Adult 9 suggested, or, as Parent 4 similarly reflected, “it looks like an old brick, and it weighs about half a kilo but it’s a prototype and I think they will be more worried about functionality in the future, once they’ve shown it works.”

In offering the above kinds of reflections which, as reported separately, led to participants offering various suggestions for how the technology could be improved for future users [19], participants, in line with their baseline accounts, also emphasised the bigger picture wherein they hoped any short-term inconveniences caused to themselves would be offset by longer-term benefits to the diabetes community. This included Adult 2 who described encountering particular difficulties using the technology due to poor eyesight and limited manual dexterity. As well as disliking, “always having to carry that big thing around in my handbag”, Adult 2 reported having been frequently disturbed by the technology at night because, “it beeped and [because of poor eyesight] I couldn’t tell if it was charging or not.” Despite the difficulties they experienced, this individual described having talked themselves out of withdrawing from the trial on the grounds that:

“the closed loop is the way forward, it’s just the equipment needs to be better … and I felt that I would let the trial down if I withdrew, because I want to do anything I can to get diabetics an easier life, and they needed to have guinea pigs that they can say they have tested it on, so they could go back and say closed loop is fantastic and can we have [regulatory] approval.”

As well as having made effort to use the prototype system, many participants described having paid greater attention to their diabetes self-management during the trial. In a few cases, as participants noted, this attentiveness had been due to the increased attention received from trial staff with whom they had had more frequent contact than in routine clinical care.  Such participants higlighted the heightened sense of accountability and motivation they had experienced as a consequence:

“it’s not every day the consultant gives out his mobile number to a patient is it? You feel like you’re quite an important patient to them at the time, you know it’s a lot of investment in one person, I did think they’re spending quite a significant investment in me and my diabetes, and I found that really nice and really motivational.” (Adult 6)

Others noted that they (or their child) had been more focused on their diabetes self-management due to the unique and privileged opportunity the trial had afforded to access cutting-edge technology: “I just felt that I’ve been given this amazing opportunity to experience something before everyone else. It made me feel really special, and that really gave me a real impetus to focus on my diabetes.” (Adult 9)

In the majority of cases, while participants were praiseworthy of the system’s ability to automatically regulate blood glucose because “it is constantly measuring it [blood glucose], and its constantly making adjustments for you” (Adult 1), they also reported having chosen to work with the closed-loop to optimize their blood glucose control; for example, by avoiding foodstuffs which caused sudden rises in blood glucose, which, as they noted, took time for the closed-loop to bring down. This included Adult 6, who, in keeping with their understanding of the trial as being “to prove it works to get it licensed” and their own desire “to have helped with that … because this trial will have implications for when the next generation of the closed-loop comes out” described how:

“I ate less things that are likely to send your [blood glucose] high, because I didn’t want to be high, it’s almost like I wanted it [closed loop] to work if that makes sense … I was quite fond of the popcorn of an evening and stuff. And sometimes I’d think: well I don’t need it. And all it’s gonna do is send me [blood glucose] up. And then it’ll [closed-loop] have to sort itself out to come back down. So I just didn’t bother.”

Adult 8, likewise, described having moderated their alcohol consumption during the trial after observing that, “when you’re drinking over and above two units you can see the effect on your blood sugar in the morning”. Adult 8 also described having avoided chocolate and other sugary snacks “as they cause my blood sugars to spike” because, as this individual explained, “I assumed they [trial team] wanted it to show in general that your HbA1c [glycated haemoglobin—a measure of average blood glucose control] has come down, and I wanted to help with that”. A similar kind of motivation and trial experience was reported by Adolescent 1, who described how, “I tried to like to keep my HbA1c down throughout the trial, I tried to control my blood sugars a lot more, to try to lower my HbA1c” because “I thought the purpose of the trial was to try and show that it [closed loop] helps the HbA1c.”

Others, including Adolescent 5, who reported how, unbeknown to staff, “for a few months I was kind of deliberately keeping it [blood glucose] a bit higher so I could get on the trial, because my blood glucose control was too good [to meet the trial inclusion criteria],” described how they had sought more input and support from staff than would have happened in a non-trial situation to help ensure they did not “mess up” the data they produced for the trial:

“I was more focused on my diabetes during the trial, I knew what I had to do and the things to kind of stay away from. Like, for instance, if it’s just your own kit and stuff, the mistakes you kind of make, you find shortcuts and things to deal with them. Whereas with the trial, you don’t want to mess up their data, so you wanted their input, you know, the ways they wanted you to deal with it.”

## Discussion

As the literature suggests, when people make decisions about taking part in trials, they weigh up the benefits to self and the benefits to others [2, 3]. In keeping with this literature, participants in the current study also presented both self-interested and altruistic agendas. In doing so, many of these individuals, who did not necessarily see themselves as future beneficiaries of closed-loop technology, also described the excitement that had resulted from taking part in a trial which had situated them at the forefront of innovation and allowed them to experience, ahead of others, a cutting-edge technology first-hand. They also shared the gratification gained from being able to present themselves to others, by virtue of their contribution to closed-loop research, as ambassadors of hope and diabetes research pioneers. In other words, for this group of individuals, trial participation could be seen as an intrinsically satisfying end in itself. Given the interwoven nature of their participation agendas, we would argue that even caveated terminology such as ‘weak’ [5] or ‘conditional’ [1] altruism does not adequately capture the complexity of these participants’ accounts. Rather, we would argue that a new concept—‘the altruselfish agenda’—needs to be introduced to recognise the potential blending and inseparability of altruistic and self-interested motivations. Indeed, in much the same way as the philosopher Badhwar has argued that altruism versus self-interest can be a false dichotomy because helping others can bolster a sense of moral self-worth [22], we would argue that contributing to a trial which was seen as ground-breaking and could potentially change lives was a very *self-satisfying* experience.

Underlying their altruselfish agendas, many participants described belonging to a wider diabetes community, one which gave rise to attendant citizenship responsibilities, including a perceived responsibility to ‘give back’ to the community by taking part in research. Such a community, as participants’ accounts further highlighted, transcended geographical and temporal boundaries, and appeared to have been generated and reinforced by their access to electronic and other media [23]. As others have noted, new communication technologies, such as the world wide web, have given rise to a new kind of citizenship in the bio-medical era—a form of digital and informational bio-citizenship, wherein erstwhile disparate individuals have come to self-identify and align on the basis of shared disease status [24]. As part of this transformative process, as these authors further suggest, bio-citizens no longer see themselves as passive recipients of health care but, rather, act together to influence research agendas in the hope of expediting better treatments for those within their ‘disease’ or ‘biological’ group [24, 25]. In this study, and in keeping with the notion of bio-citizenship, participants appeared both to self-identify with a wider diabetes community which had attendant citizenship responsibilities and to perceive themselves as being co-researchers and co-producers of knowledge who, by virtue of their trial involvement, could champion and support the progression of closed-loop technology. By drawing attention to this bio-citizenship role, our study brings a new and important question for trial (and potentially other clinical) research to the fore; namely, if individuals do have an intellectual and personal stake in the research process, and if they really want an intervention to work, might this influence how they conduct themselves during a trial and, hence, potentially its outcomes?

The idea that trial participation can lead to positive clinical outcomes which may be absent or less apparent in routine clinical care has already been considered by others. Indeed, various researchers have reflected upon the possibility that trial participation, by its very nature, might result in improved clinical outcomes independent of whether the intervention being trialled is more efficacious than the control or treatment offered in routine clinical practice [26]. This phenomenon, which has been termed the ‘trial effect’ or ‘side effect’ of a trial [27], is thought to be due to the possibility that, amongst other things, trial participants may experience a positive care effect as a result of the extra contact and follow-up support received from staff [27]. It has also been suggested that trial participants (and staff) may change their behaviour during a trial because of feeling and/or being under close observation [26], an issue which has been termed the ‘Hawthorne effect’ [27, 28]. As the findings of two key reviews suggest, while the idea of a trial effect seems plausible, there is currently only weak evidence for its existence, an issue which, as the authors note, may be due to difficulties measuring and quantifying this phenomenon [27, 29]. While these authors highlight methodological challenges to measuring and determining trial effects, the absence of studies, such as our own, drawing directly on trial participants’ own understandings and experiences also needs to be noted.

Our study, with its longitudinal design, allowed us to explore whether participants’ motivations and agendas for taking part did, potentially, influence how they conducted themselves during the trial. To this end, and in keeping with their initial motivations and understandings, participants not only described making effort to use what was seen as bulky and cumbersome prototype technology, many also reported being more attentive to, and focused on, diabetes management by virtue of being in the trial. As some individuals noted, this extra effort had been made because they had felt more accountable to trial staff with whom they had more frequent contact than in non-trial situations; indeed, some noted that this desire for increased accountability had influenced their decisions to take part. While this observation supports current understandings of a “trial effect”, our analysis has also drawn attention to how participants’ motivations for taking part and, relatedly, their intellectual and emotional investment in progressing closed-loop technology, also requires consideration. Specifically, and in line with the altruselfish agendas which had driven their participation, participants described making effort during the trial and having been more focused on their diabetes management. As these participants suggested, they had done this in order to help create the conditions in which the closed-loop system could work most effectively to optimize their blood glucose control, this being one of the trial’s primary endpoints.

In line with interview participants’ expectations, understandings and agendas, the main trial results have shown that individuals in the closed-loop arm did experience a significantly greater reduction in HbA1c levels compared to the control group [30]. While the clinical efficacy of the technology has thus been demonstrated in this and a diversity of other trials of closed-loop systems [31], we have highlighted the importance of considering participants’ own agendas and agency when interpreting the results of such trials. In taking our observations and arguments forward, however, readers may wish to consider that our research involved individuals who did not need the closed-loop to manage their diabetes and keep themselves alive. In other words, we might have accessed a very different kind of trial participant to those in third world countries and/or who cannot access or afford (private) health care, amongst whom an altruselfish agenda might not be so keenly felt [32]. The relatively small-scale nature of the trial, and some of the closed-loop research which proceeded it, may also need to be taken into account; specifically, in terms of how this might have fostered a perception and understanding that participants’ own effort and data could potentially have a tangible impact on the trial’s outcomes. Hence, future work could consider the perspectives and experiences of those involved in larger-scale (diabetes) trials, including trials where blinding is possible and, hence, participants do not know which treatment arm they are in. While we only considered the perspectives of individuals randomized to the closed-loop arm, our study raises important questions about how people might react to being randomized to a control arm. For instance, do such individuals behave differently due to disappointment, a competitive element (and hence, potentially, a desire to beat those in the intervention arm), or an on-going desire to help prove the efficacy of a trial intervention by trying less hard? Only limited insight can be gained from other studies which have explored the reactions and experiences of control group participants. Lindström et al. [33], for instance, consulted individuals in the control arms of two smoking cessation trials. These authors not only found disappointment to be commonplace but also that, potentially, this led to higher drop-out rates. Petersen et al. [34] also found disappointment to be the pervading reaction amongst parents of newborns who were randomized to the control group in a trial of the Bacillus Calmette-Guérin (BCG) vaccine at birth. However, given this trial only involved a one-off intervention—an injection which newborns in the control arm did not receive—it is not possible to determine the implications of disappointment for subsequent trial conduct.

## Conclusions

To date, most work seeking to understand whether, and why, an intervention might work differently in a trial than in routine clinical care has drawn upon the understandings and experiences of trial staff. In doing so, this work has offered important insights into why protocols and procedures are not always followed in trial contexts [15, 35–38]. As a complement to this work, our study highlights the importance of considering trial participants’ own understandings, motivations and agendas for taking part. Specifically, our study raises important questions about how trial results should be interpreted in an era where, increasingly, patients (bio-citizens) are not only driving research agendas [24] but may also see themselves as co-researchers and co-producers of knowledge who have an active and personal investment in the outcomes of (trial) research.

## Abbreviations

BCG: Bacillus Calmette-Guérin
HbA1c: Glycated haemoglobin
